# Collective behavior of thermophoretic dimeric active colloids in three-dimensional bulk

**DOI:** 10.1140/epje/s10189-021-00043-8

**Published:** 2021-03-27

**Authors:** Martin Wagner, Sergi Roca-Bonet, Marisol Ripoll

**Affiliations:** Theoretical Physics of Living Matter, Institute of Biological Information Processing, Forschungszentrum Jülich, 52425 Jülich, Germany

## Abstract

**Abstract:**

Colloids driven by phoresis constitute one of the main avenues for the design of synthetic microswimmers. For these swimmers, the specific form of the phoretic and hydrodynamic interactions dramatically influences their dynamics. Explicit solvent simulations allow the investigation of the different behaviors of dimeric Janus active colloids. The phoretic character is modified from thermophilic to thermophobic, and this, together with the relative size of the beads, strongly influences the resulting solvent velocity fields. Hydrodynamic flows can change from puller-type to pusher-type, although the actual flows significantly differ from these standard flows. Such hydrodynamic interactions combined with phoretic interactions between dimers result in several interesting phenomena in three-dimensional bulk conditions. Thermophilic dimeric swimmers are attracted to each other and form large and stable aggregates. Repulsive phoretic interactions among thermophobic dimeric swimmers hinder such clustering and lead, together with long- and short-ranged attractive hydrodynamic interactions, to short-lived, aligned swarming structures.

**Graphic Abstract:**

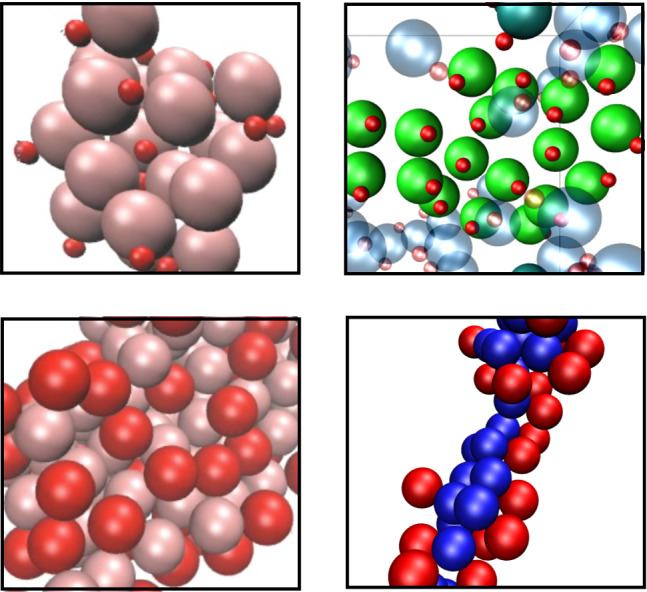

## Introduction

Systems with at least one component able to transform energy into motion are referred to as *active matter* [1]. These systems are ubiquitous in biological systems and have components ranging from the meter scale of sheep or birds to micrometer scale of bacteria or algae, or even to the nanometer scale of sub-cellular structures such as microtubules. Synthetic active systems, especially with components in the micrometer scale, are currently a subject of great technological interest due to the development of new bio-mimetic materials [2, 3]. Artificial microswimmers with a propulsion mechanism based on phoretic effects [4, 5] can behave like passive colloids when unless they are thermally [6–8], chemically [9–12], or electrically activated [13, 14], which can provide very interesting possibilities to these materials. In particular, thermophoretic swimmers [6, 15, 16] consist of solutions of colloids whose surfaces are composed of or coated with two materials. These two materials have very different absorption coefficients, like gold and silica, such that environmental heating can produce a steady local temperature gradient around the colloid. This produces a thrust on the colloid causing the persistent self-propulsion of the whole swimmer. Thermophoretic swimmers are therefore easily bio-compatible since they can be powered without any chemical modification of the solvent. Large versatility is expected from devices based on this effect since thermophoresis has shown to be very sensitive to a large number of factors like pressure, average temperature, or solvent composition [17–19] and also since the heat sources, mainly lasers but also magnets, can be very precisely controlled in time and space [20, 21].

When many phoretic colloidal swimmers come together, their dynamic collective behavior shares many properties with other systems of active particles, but also displays specific and varied characteristics. Chemically active Janus colloidal particles have already shown clustering and self-assembled structures [22–24] as well as schooling behavior, and the formation of living crystals has already been observed for light-powered micromotors [25–27]. Interestingly, simulations of thermophobic dimers have recently shown the existence of a dynamic swarming behavior with front-like propulsion which occurs due to the specific combination of axial propulsion, phoretic repulsion, and hydrodynamic interactions [28]. There already exist various methods to perform simulations of active matter which account for the effects of propulsion, thermal fluctuations, hydrodynamics, and phoretic interactions [29–33]. However, most of these models neglect one or various of the previous contributions or consider them as independent from each other [34, 35]. Due to the intrinsic details of the phoretic mechanism, it is impossible to independently tune propulsion, inter-swimmer interactions, and hydrodynamic interactions, such that their theoretical investigation requires the development of specific models. Hydrodynamic interactions have already shown to affect the phase behavior of systems of squirmers [36, 37], and although in many cases the effect of short-range hydrodynamic interactions is not considered, it has also been shown that they can have an important influence [38]. In our simulation approach, shape and strength of phoretic and hydrodynamic interactions are not directly imposed but a consequence of the interactions of the solvent and the colloid at the bead surface.

In this work, we extend the study of thermophobic dimeric swimmers [28] to further investigate various dimeric geometries and densities for thermophobic dimers and now also for thermophilic dimers. Thermophilic active dimers form ordered, stable, and static clusters. In contrast, thermophobic active dimers might form short-lived oriented clusters, with structures that depend on both long- and short-range hydrodynamics. Although here we concentrate on the case of thermophoresis, the discussed concepts can be extrapolated almost straightforwardly to the case of diffusiophoresis. Concentration gradients are typically the consequence of a localized chemical reaction, and due to the same nature of density and thermal gradients, the diffusion equations have the same form.

## Simulation model

The employed method considers a hybrid approach which combines a hydrodynamic solvent simulated via multiparticle collision dynamics (MPC) with a coarse-grained description of the colloids. The colloid–colloid and the colloid–solvent interactions are performed with molecular dynamics (MD) [39, 40]. In MPC, the solvent is accounted for by a large number of explicit point particles of mass *m* which perform alternating streaming and collision steps. In the streaming step, particles propagate ballistically during the so-called collision time *h*. In the collision step, particles are sorted into cubic boxes of side *a*, with origin being chosen following a grid shifting procedure [41]. We use the stochastic rotation dynamics collision, where each particle interchanges momentum with other particles within the same cell by rotating the velocity relative to the center-of-mass velocity of the cell around a random axis by an angle $\alpha$. The choice of $a=1=m=k_\mathrm {B}\overline{T}$ defines the simulation units, so that time is scaled with $(ma^2/k_\mathrm {B}\overline{T})^{1/2}$ and velocity with $(k_\mathrm {B}\overline{T}/m)^{1/2}$. Other parameters here chosen are $\alpha =120^\circ$ and $h=0.1$, together with the average number of particles per collision cell, $\rho =10$. These numbers determine the fluid transport properties as the diffusion coefficient $D_\mathrm {s}=0.06$, the kinematic viscosity $\nu =0.79$, or the thermal diffusivity $k_T=0.15$ [42–44]. The resulting Schmidt number, $\mathrm {Sc}=\nu /D_\mathrm {s}=13$, is smaller than that of water, but shows that the propagation of momentum is faster than that of mass, which has extensively been shown to provide a very efficient approach to include hydrodynamic interactions [45, 46]. The related Prandtl number, $\mathrm {Pr}=\nu /k_T=5.3$, is very close to that of various fluids such as water and, most importantly, enables local temperature gradients to remain stable under adequate boundary conditions. Furthermore, this description has shown to properly incorporate hydrodynamic interactions when applied to many different colloidal, polymeric, and biological systems [47, 48], and in particular in phoretic systems [49–51].

**Table 1:** Single swimmer properties calculated for two geometries of both thermophobic and thermophilic dimers

	Phob $\gamma {=}1$	Phob $\gamma {=}3$	Phil $\gamma {=}1$	Phil $\gamma {=}3$
$v_\mathrm {s}$	$-\,0.021$	$-\,0.020$	0.014	0.013
$D\, (\times 10^{4})$	6.29	11.5	9.61	14.1
$D_\mathrm {r}\, (\times 10^{5})$	2.92	9.89	4.57	24.6
$\mathrm {Pe}$	120	34	51	8.8

**Fig. 1:**
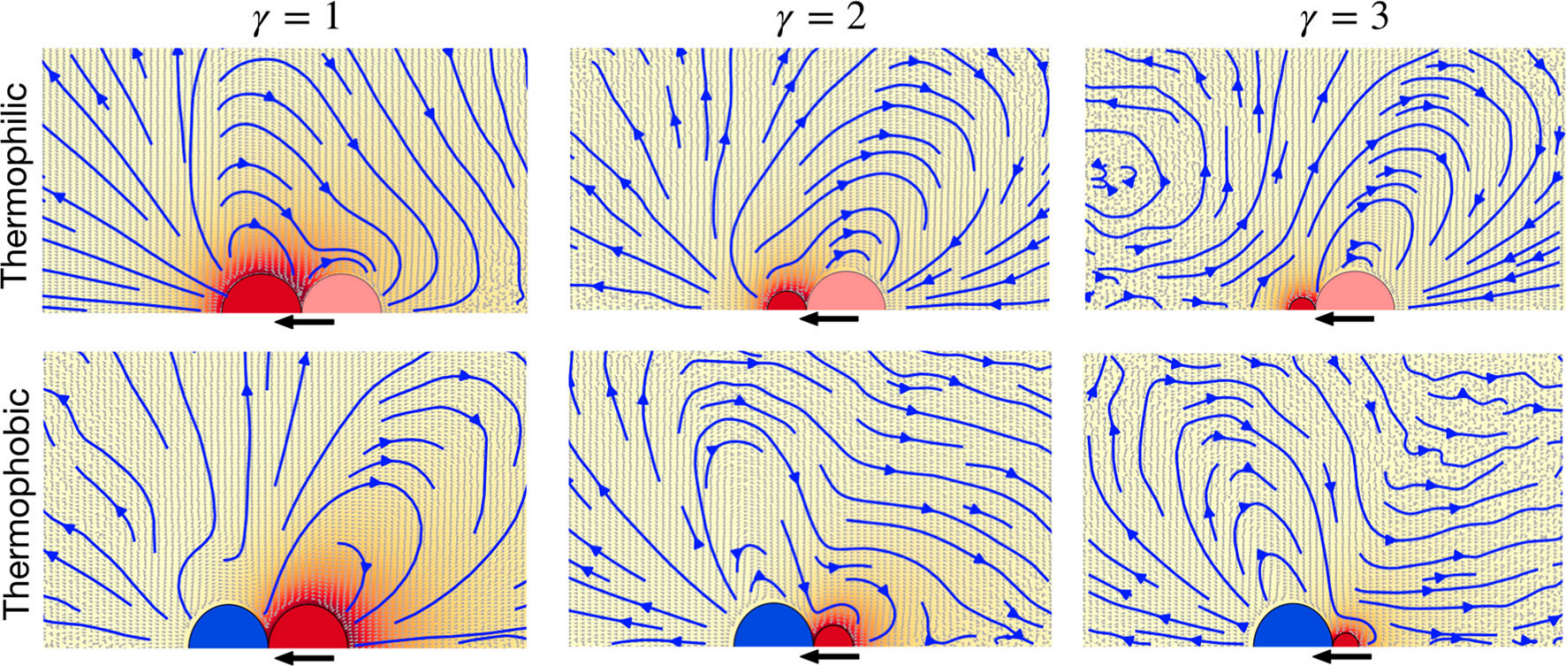
Flow fields in the areas close to the thermophilic dimer for the three considered geometries: Symmetric with $\gamma =s_p/s_h=1$, and asymmetric with $\gamma =2$, and $\gamma =3$. Hot beads are colored red, thermophilic phoretic beads light pink, and thermophobic phoretic beads blue. Solid blue lines denote the stream lines, light gray arrows are the flow velocities, and the background color stands for the temperature field. Solid black arrows under the dimer indicate the swimming direction. Due to symmetry, only half of each colloid is shown

The phoretic character of the colloids (thermophobic or thermophilic) is determined by the colloid–fluid interactions which are here modeled via MD. We use displaced Mie-like potentials [52, 53] given by

1 $$\begin{aligned} U(r) = 4 \epsilon \left[ \left( \frac{\sigma }{r - \varDelta } \right) ^{2n} - \left( \frac{\sigma }{r - \varDelta } \right) ^{n} \right] + C, \end{aligned}$$

where *r* is the pairwise distance, $$\epsilon $$ describes the strength of the potential which we choose as $$\epsilon =k_\mathrm {B}\overline{T}$$, and *n* determines the potential softness. The additional displacement introduced by the parameter $$\varDelta $$ permits to independently vary the colloid size and the depletion layer [54]. With this model, the radius of each bead is $$s\equiv \sigma +\varDelta $$, and we denote the related size parameters as $$(s,\varDelta )$$. Repulsive interactions here are simulated with $$C=\epsilon $$, $$n=24$$ and $$r_\mathrm {c}=2^{1/n}\sigma +\varDelta $$, and attractive with $$C=0$$, $$n=3 $$ and $$r_\mathrm {c}=1.13\sigma +\varDelta $$. The use of a repulsive colloid–solvent potential has shown to result in a thermophilic behavior, while attractive interactions translate into a thermophobic behavior [55]. Dimers investigated here are constructed by one hot bead with repulsive interactions of size $$s_\mathrm {h}$$, which we fix as (6, 3), (3, 1.5) or (2, 0.5); and one non-heated bead with size $$s_\mathrm {p}$$, which we fix as (6, 3) throughout this work. We refer to the non-heated bead as the phoretic bead, since it experiences a thrust due to the temperature gradient in the surrounding solvent. Both beads are held together by a strong harmonic potential at a distance $$s_\mathrm {h}+s_\mathrm {p}$$. Mimicking the heating obtained by laser illumination of partially gold-coated particles [6], we rescale the temperature of fluid particles in a short layer ($$0.08 s_\mathrm {h}$$) around the hot beads to a value of $$T_\mathrm {h}=1.5$$, while keeping the overall average fluid temperature at $$\overline{T}=1.0$$ using simple velocity rescaling [7, 44]. This constant heating neglects shadowing effects [31] which is not expected to have large influence, as later discussed. Simulations are performed using a modified variant of the software package lammps [56], in particular a modified version of the “srd”-package [57]. The time step to integrate the potential interactions is $$\varDelta t = 0.01 h$$, and the bead mass, *M*, is chosen such that the swimmers are neutrally buoyant.

## Single dimeric properties

The phoretic bead radius $s_\mathrm {p}$ and the ratio between the phoretic and the hot beads radius $\gamma =s_\mathrm {p}/s_\mathrm {h}$, together with the fluid properties, and the magnitude of the applied temperature gradient, have shown to determine the single dimer velocity $v_\mathrm {s}$ and the rotational diffusion coefficient $D_r$. The corresponding Péclet number can then be calculated considering a dimer relevant length scale for which we chose $s_\mathrm {p}$, the phoretic bead radius, such that $\mathrm {Pe} = v_\mathrm {s} / (D_\mathrm {r} s_\mathrm {p})$. Other choices of the relevant distance are possible, we chose $s_p$ since it importantly influences the strength of phoresis-related interactions, and it is fixed in our simulations. Swimmers with larger values of $\mathrm {Pe}$ will display more straight trajectories than swimmers with smaller values of $\mathrm {Pe}$. These single particle properties for the reference dimers here investigated are summarized in Table 1, where, for completeness, we also calculate the dimer translational diffusion coefficient *D*. Equilibrium simulations quantifying the mean angular and linear squared displacements have been used to determine $D_r$ and *D*, while simulations with the here chosen value of $T_h$ are made to determine $v_s$. For the same dimers, other values of $\mathrm {Pe}$ can be achieved simply by changing the temperature of the hot dimer $T_h$, although here we consider this parameter fixed.

Colloid shape has shown to importantly affect the phoretic particle properties [58–60], and also the induced flow field. Therefore, in the case of active phoretic dimers, the direction, shape, and intensity of the induced solvent velocity are determined by the surface properties of the swimmers and by their overall geometry [7, 28, 61]. Flow fields and stream lines around various thermophoretic dimers are shown in Fig. 1. Results in these figures are calculated for single swimmers in the co-moving dimer frame, in a cubic box of size $L=10(s_\mathrm {p}+s_\mathrm {h})$. Flow velocities are calculated by a cylindrical spatial average, which is then averaged over 20 simulations, each for $10^4$ MPC time units. Thermophilic symmetric dimmers, $\gamma =1$, show axial front stream lines departing from the swimmer, which translates into a hydrodynamic repulsion with any other particle placed in that area. Laterally, the stream lines are directed to the swimmer which translates into a hydrodynamic attraction with other neighboring particles. This is consistent with the behavior of a hydrodynamic pusher [62]. When changing the swimmer geometry, the hydrodynamic flow field adapts to the size ratio of the constituent beads, and when the hot bead decreases in size, the repulsive front velocity displaces progressively to the lateral region, such that the hydrodynamic field is more consistent with that of a puller, as can be seen for the thermophilic dimers with $\gamma =2,3$ in Fig. 1. Thermophobic swimmers show a behavior reciprocal to that of thermophilic swimmers, as evident in Fig. 1, while symmetric dimers display a lateral repulsion typical for pullers, and asymmetric dimers display a lateral attraction typical for pushers. In this case, the axial back stream lines are attractive in the symmetric case, which seems to displace to lateral front areas in the asymmetric cases. It is very important to note that none of these flow fields correspond to the ideal cases of pullers or pushers, but they are clearly less symmetric and much more intricate. We emphasize also that we do not directly impose any particular hydrodynamic behavior, but obtain it by the accommodation of the employed hydrodynamic explicit solvent method to the colloid surface properties which are different in each case, similar as it would experimentally occur.

**Fig. 2:**
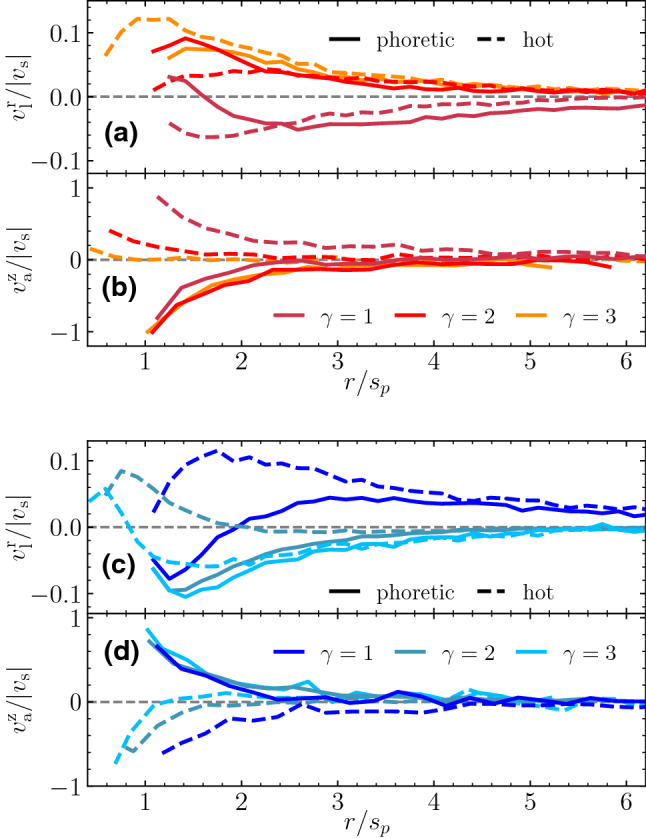
**a**–**d** Average normalized velocity of the fluid as a function of the distance from the bead center, as extracted from the data presented in Fig. 1. The velocity is normalized with $|v_s|$, the single swimmer velocity for each geometry. The distance is expressed in units of the phoretic bead radius. **a**, **c** Velocity in the radial direction in two parallel axes starting on each of the two beads center and in the direction perpendicular to the dimer axis. **b**, **d** Velocity in the axial direction with positive direction toward the dimer. In both plots, the three geometries are colored as indicated in the labels, solid lines correspond to the axis starting at the phoretic bead, and dashed lines to the axis starting at the hot bead. **a**, **b** correspond to thermophilic swimmers; **c**, **d** to thermophobic swimmers

A quantitative characterization of the flow velocities is presented in Fig. 2 for the different types of thermophoretic dimers in two perpendicular axes for each bead, namely the direction perpendicular and along to the dimer axis. Since the distances are considered starting at the center of the beads, positive values of the velocities account for flows going away from the bead, namely repulsive interactions; while negative velocity values indicate fluid flows going toward the bead, this is attractive interactions. Figure 2a shows that thermophilic symmetric dimers, $\gamma =1$, are laterally attractive, while asymmetric ones, $\gamma =2,3$, are laterally repulsive. In the front axial direction, they are all attractive, as can be seen in Fig. 2b, which act together with the phoretic attraction. Thermophobic symmetric dimers are laterally repulsive, as shown in Fig. 2c, although there is an area close to the phoretic bead where there is an attractive hydrodynamic interaction. Meanwhile, asymmetric thermophobic dimers ones are attractive in the front axial direction and repulsive in the rear axial direction. It can be seen that the main features of the thermophilic and thermophobic swimmers can be understood as being reverse of each other, but the flows and the velocity values clearly differ from an exact reverse version.

**Fig. 3:**
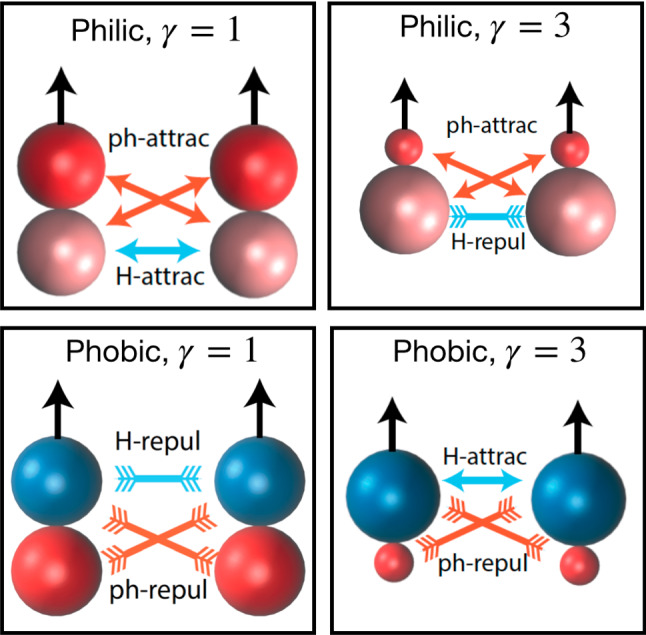
Sketches of the hydrodynamic and phoretic pair interactions between thermophoretic swimmers

The above discussed hydrodynamic interactions have to be considered together with the intrinsic phoretic interactions which are also attractive or repulsive. In Fig. 3, the interactions in the four most representative cases are sketched. Thermophilic symmetric dimers show to have both interactions attractive, while thermophilic asymmetric dimers combine phoretic attraction with hydrodynamic repulsion. Thermophobic symmetric dimers show to have both interactions repulsive, while thermophobic asymmetric dimers combine phoretic repulsion with hydrodynamic attraction. Besides phoretic and hydrodynamic interactions, the effect of self-propulsion, excluded volume interactions, and thermal fluctuation will also importantly influence the collective behavior of the dimeric swimmers.

## Thermophilic collective properties

**Fig. 4:**
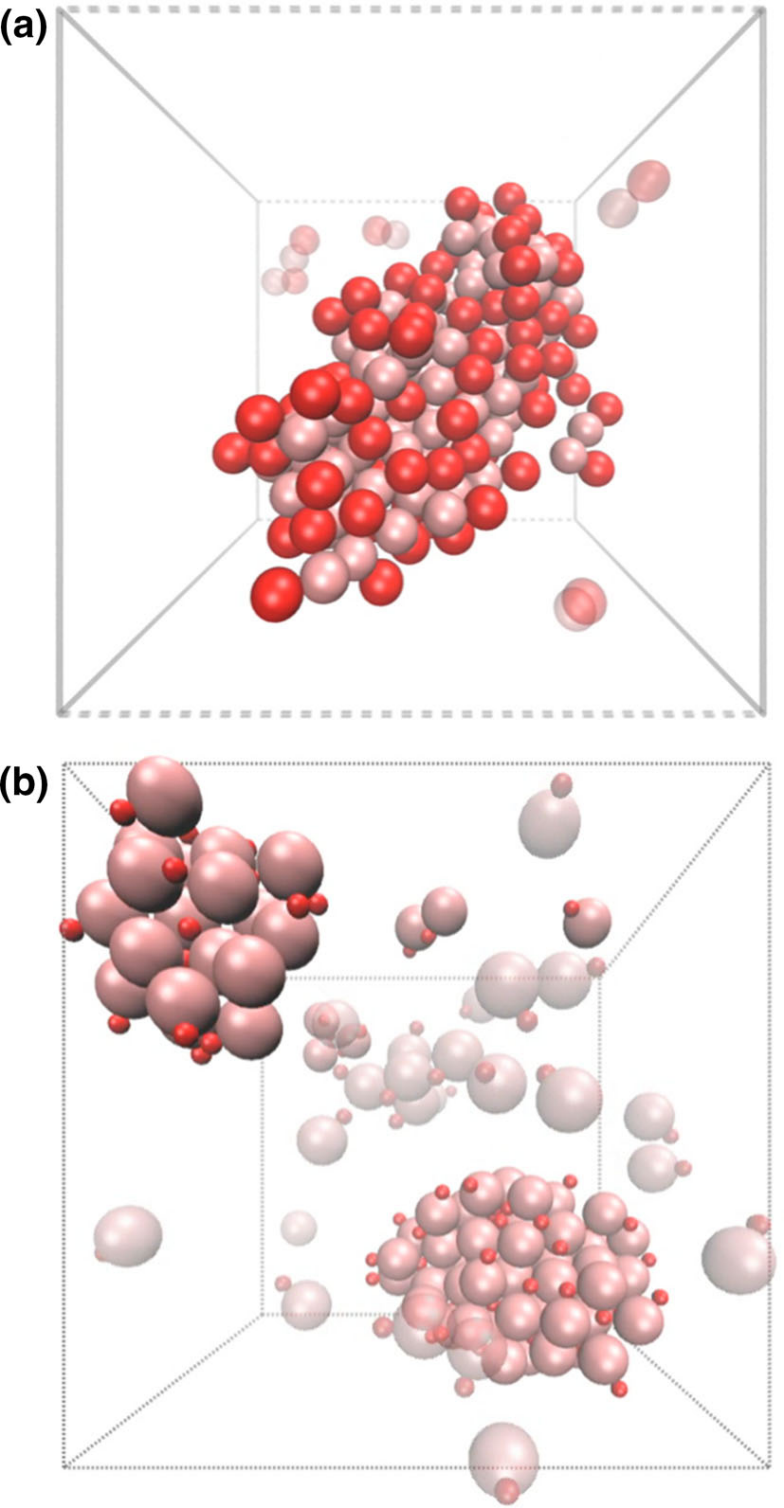
Snapshots of a system with 100 thermophilic swimmers at volume fraction $\phi =0.05$ for a) the symmetric $\gamma =1$ and b) asymmetric $\gamma =3$ cases. Unbounded moving dimers are depicted translucent, clustered dimers are colored solid, and lines refer to the periodic boundary simulation box. Hot beads are colored bright red, and phoretic beads light pink

Simulations in the collective regime show that thermophilic dimers freely propel with the hot bead at the front, until they get close enough to other swimmers, moment at which they mostly remain together. This occurs for both symmetric and asymmetric dimeric swimmers (see Fig. 3), in spite of the repulsive lateral hydrodynamic interactions and due to the attractive phoretic interactions and re-arrangement of the resulting temperature field, as later discussed. Free dimers colliding with a previously nucleated cluster make the cluster slowly increase in size. For the parameters checked in this investigation, this process is expected to finish in a completely collapsed state. The time for the free dimers to aggregate to the nucleated cluster increases with decreasing density. This also means that a nucleated cluster does not grow linearly with time, and that the final collapse can be, in many cases, just an asymptotic state. Figure 4 presents snapshots of two systems with symmetric and asymmetric thermophilic swimmers after *ca.* $7\times 10^5$ MPC time steps. These clusters of self-phoretic thermophilic dimers do not collectively propel and do not rotate either, moving only like a bigger Brownian entity. When several clusters are formed, these eventually coalesce, although depending on the overall density, the time for this to occur can be large. The finite simulation time is therefore the reason for the few free particles in both Fig. 4a, b and the two well-separated clusters in Fig. 4b. Although the main behavior is similar for dimers of the two symmetries, we can observe that clusters of asymmetric dimers are more spherical than the clusters of symmetric counterparts.

From the snapshots in Fig. 4, it can be clearly seen that the dimers at the cluster surface have the hot bead pointing outwards. Thermophilic dimers move with the hot bead at front; however, when placed at the cluster surface, they do not just swim away, but remain attached to the cluster. This is in contrast to clusters of various experimental and theoretical model active systems in which swimmers aggregate with the propulsion direction of the particles pointing inwards [63–65]. This shows that the clustering in these thermophoretic dimers is not induced by jamming or motility-induced attraction, as these steric effects would not hinder a dimer pointing outwards from just swimming away. The main point is that phoretic swimmers do not stop their motion only due to steric interactions, but also due to other mechanisms. First, two oppositely oriented phoretic swimmers get attracted to each other, and the resulting bounded pair does not significantly move, since the driving forces cancel. Similar construction also occurs in the case of larger aggregates of ordered dimers. Second, when one phoretic bead is surrounded by various other hot beads, the temperature distribution is much more homogeneous, which diminishes all interactions, and these are the self-propulsion, the hydrodynamic interactions, and the inter-dimer phoretic attractions. Therefore, for a dimer at the cluster surface we have that the hot bead does not react to any exterior temperature field and the phoretic bead of the swimmer will be drawn toward the hot beads of other dimers. If the temperature field is then uniform enough, the driving force is very small, and the dimer stays as part of the cluster, although its front bead is pointing outwards. Furthermore, this strongly diminished propulsion of the dimers inside the cluster is also the reason for the clusters lack of rotation and propulsion.

On the other hand, it is to be noted that disregarding shadowing effects here considered is not expected to be a too significant approximation, due to the homogeneous temperature within the cluster, although it could be that in the case of directed heating, the colder side of the cluster might be less compact. This would give rise to some partial dissemble rate, with distortion of the cluster shape and reduction of its size, which would not resemble the described comet-like behavior [31]. Also strongly related are the simulations results of chemically powered dimeric colloids in quasi two-dimensional confinement [66, 67], which also show the formation of clusters of phoretic dimers, although in that case the dimers at the surface of the formed clusters are oriented toward inside the cluster. To more precisely distinguish the role of phoretic and hydrodynamic interaction in the different cases requires the development of specific phoretic Brownian models. This is the subject of current research and will be reported elsewhere.

**Fig. 5:**
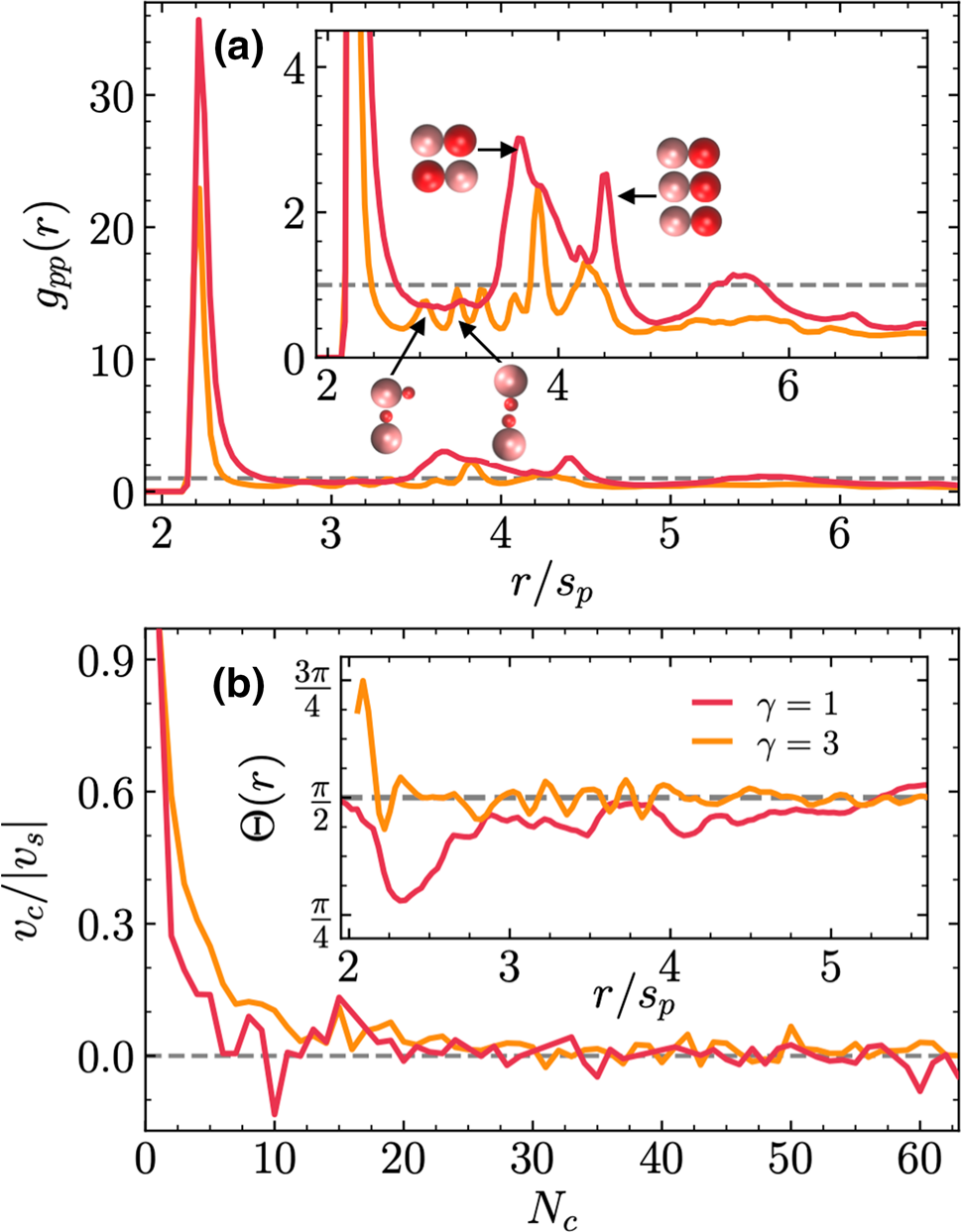
Quantitative analysis for the thermophilic dimers in Fig. 4. **a** Phoretic–phoretic beads pair correlation function; the inset is a zoom in the peaks area, with sketches of the corresponding typical pairs configurations. **b** Normalized averaged velocity as a function of the cluster size. Inset in **b** averaged pair orientation as a function of the relative distance

Structural properties of the clusters can be quantitatively analyzed directly from the simulation data and by further averaging over time and different realizations, typically 3 to 5, at a later stage of the simulation, where the clusters are already formed and mostly stable. The pair correlation function calculated for pairs of phoretic beads $g_{pp}(r)$ is displayed in Fig. 5a, where the largest peak is shown for particles at contact. At larger distances, numerous peaks appear as a signature of the crystal-like structure of the clusters. Symmetric swimmers show several superimposed peaks around $r=4s_\mathrm {p}$. The peaks for distances smaller than $4s_\mathrm {p}$ correspond to close dimers with opposite orientations, and the peaks for distances a bit larger indicate a second coordination layer one bead farther away, as sketched in the inset of Fig. 5a. Asymmetric swimmers show several secondary peaks at much closer distances which correspond to various configurations with one or several of the smaller hot beads in between the phoretic beads. We also calculate $\varTheta (r)$, the average orientation of dimer pairs as a function of their separation, which is shown in the inset of Fig. 5b. Asymmetric dimers show orientations always very close to $\pi /2$ which is the average value of all possible orientations. This indicates that there is no net alignment of the dimer orientations inside the cluster, which is due to the partial symmetric crystalline order, partial random orientation of the dimers inside the cluster. Symmetric dimers show average orientations smaller than $\pi /2$, especially at shorter distances, which indicates that dimers are more frequently aligned in the same direction. This preferred alignment does not persist for longer distances since that would result in an overall propulsion of the cluster which Fig. 5b indicates does not occur. The difference in the pair orientations for the two dimer symmetries can also be related with the overall shape difference of the cluster, close to spherical for $\gamma =3$ and more elongated for $\gamma =1$ as shown in Fig. 4. The dimer and cluster dynamics can be characterized by considering the cluster velocity $v_\mathrm {c}$, *i.e.*, the average velocity of the dimers projected on the cluster orientation, as a function of cluster size, as shown in Fig. 5b. While clusters with a small number of bonded dimers might display some random motion with an effective non-vanishing cluster velocity, larger clusters show to have clearly vanishing velocities. Important to note is that all simulation results here shown correspond to a fixed large value of the applied temperature difference, and two particular values of the thermophoretic strength, here given by the shape of the potentials, and experimentally determined by the colloid and solvent properties. Different temperature differences and colloid–solvent properties will display larger or smaller dimer velocities and weaker or stronger interactions, which are expected to remain qualitatively the same.

## Thermophobic collective properties

Simulations of thermophobic swimmers in the collective regime show that they propel with the hot bead at the back, and that instead of the assembly of stable compact clusters, short-lived aligned swarming structures are formed.

**Fig. 6:**
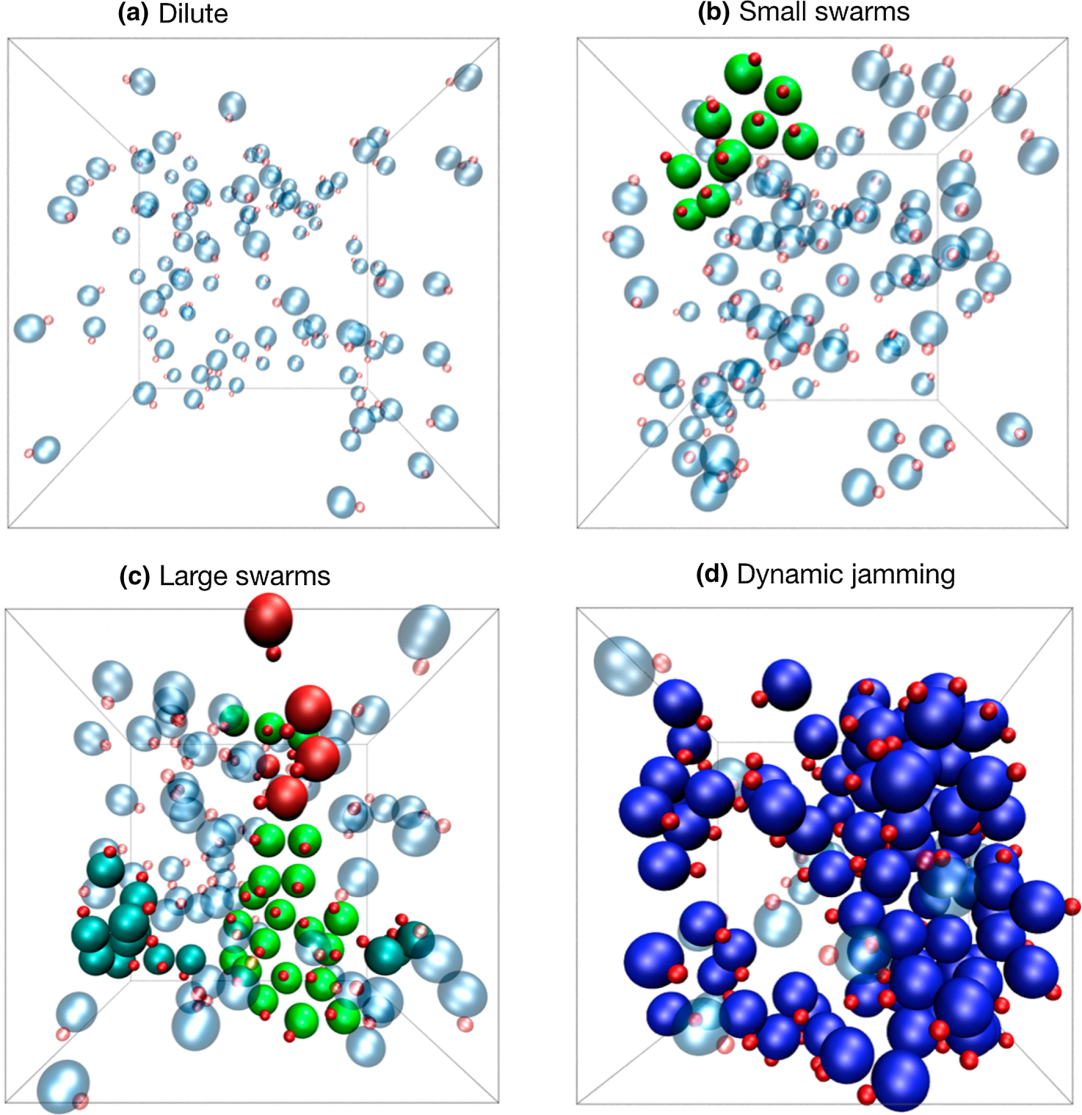
Snapshots for typical configurations of ensembles of $N=100$ thermophobic dimers of bead size ratio $\gamma =3$, for four volume fraction values, which correspond to four different association states. **a** $\phi =0.010$ corresponds to the dilute regime, **b** $\phi =0.025$ to the small swarming regime, **c** $\phi =0.050$ to the large swarming regime, and **d** $\phi =0.100$ corresponds to the dynamical jamming regime. Hot beads are red colored, freely swimming dimers have the phoretic bead blue-translucent colored, and dimers belonging to a cluster with five or more dimers have the phoretic bead solid bright colored. Different clusters can be distinguished by color, since all dimers within one cluster have the same color

To characterize the collective behavior, we perform simulations for various values of the volume fraction in each of the three considered dimer geometries. The investigated systems can be classified in four different regimes, which are illustrated in Fig. 6 for the asymmetric case with $\gamma =3$.

**Fig. 7:**
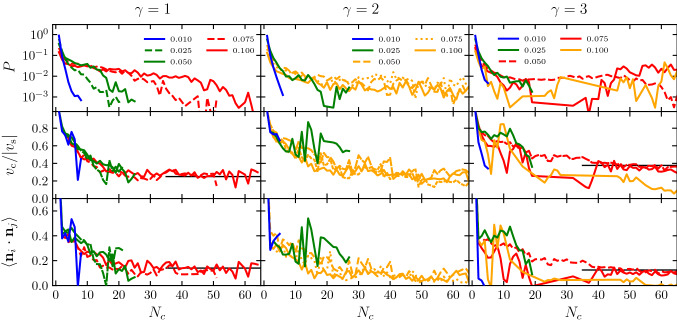
Quantitative cluster analysis for systems of thermophobic dimers with three bead size ratios, $\gamma =1,2,3$, at different densities $\phi$, as stated in the labels. *P* is the normalized probability of a dimer to be in a cluster with $N_c$ dimers. $v_c/|v_s|$ is the average center of mass velocity $v_c$ of clusters with size $N_c$, normalized by $|v_s|$, the single dimer velocity. $\langle \mathbf{n}_i \cdot \mathbf{n}_j \rangle$ is the average correlation of the dimer orientations within each cluster. Line colors refer to the dynamical regime, consistent with the colors in the dynamical state diagram in Fig. 8, and black underlying solid lines are a guide to the eye to indicate average cluster velocities and orientations of the large swarm state

To distinguish, to characterize, and to provide a quantitative description of these states, we perform a cluster analysis as shown in Fig. 7. Clusters of dimers are identified by applying a cluster criteria. Here, two dimers are considered to belong to the same cluster if their beads *i*, *j* are closer than $1.32(s_i+s_j)$, for times longer than 300 MPC units of time. The considered distance is $10\%$ larger than the colloid–colloid minimum interaction distance, and the time is also a bit larger than the time a dimer takes to displace its own phoretic bead radius. Clusters are then classified by the number of constituent dimers $N_c$. Figure 7 shows the probability distribution of the different cluster sizes, as well as the cluster velocity, and the cluster orientation. The measured *P* is calculated as the probability of a dimer being in a cluster of size $N_c$. The number of dimers in each cluster fluctuates rapidly and strongly since dimers constantly attach and detach, and the clusters are easily dividing, merging, or simply colliding with other clusters. Therefore, valuable indications of the typical cluster sizes can be made with averaged values of *P* with $N_c$, plateau values, and with the maximum $N_c$ values at which *P* is still significant. The cluster velocity $v_c$ is the magnitude of the average velocity of all dimers in the cluster $\mathbf {v}_c = \langle \mathbf{v}_i\rangle$. Finally, the average cluster orientation is characterized by the average orientation correlation $\langle \mathbf{n}_i \cdot \mathbf{n}_j \rangle$, with $\mathbf{n}_i$ and $\mathbf{n}_j$ the orientation of all pairs of dimers belonging to the same cluster. Simulations are *ca.* $7\times 10^5$ MPC time steps, and averages in Fig. 7 disregard the first $10^5$ steps.

The physical mechanisms determining the system dynamical behavior can be understood as a result of the combination of phoresis, hydrodynamic interactions, and thermal fluctuations. The flattened structures appearing at intermediate densities are due first to the strongly axial repulsion. In the front, repulsion is due to the hydrodynamic flow-mediated interactions (see Fig. 1), and in the back, repulsion is due to phoresis. Two dimers placed side by side get aligned due to the phoretic interaction and stabilized due to the lateral hydrodynamic attraction. The clusters with a reasonably high degree of alignment propel then collectively, with a velocity $v_c$ smaller than that of the single swimmer, which varies with the cluster size and overall conditions. The swarming fronts are then destabilized by thermal fluctuations and random collisions with other clusters. We discuss now the observed regimes for systems of asymmetric dimers $\gamma =3$, as shown in the snapshots in Fig. 6 and the analysis in Fig. 7. When the volume fraction is very low, i.e., $\phi =0.01$, as shown in Fig. 6a, the small structures that eventually build up are dissolved by thermal fluctuations before more dimers can assemble, such that the dimers move effectively free and only a few very small collision clusters can be detected with our cluster analysis in Fig. 7. Therefore, we refer to this as the *dilute regime*. When the volume fraction is slightly larger, $\phi =0.025$ in our case, a few more swimmers are attached in a moving front, but the clusters do not significantly grow. For the studied density in Figs. 6b and 7, these clusters are made of typically 15 dimers and never more than 20. Cluster velocities and orientational correlations are reasonably high, such that we refer to this as the *small swarms regime*. At larger volume fractions, here $\phi =0.05$, and 0.075, these clusters can be become much larger, up to $60-80$ dimers for simulations with a total 100 dimers, and can reach up to 200 dimers for simulations with 500 dimers (not shown here, see Ref. [28]). The orientational order is clearly non-vanishing, and the cluster velocities are also close to $40\%$ the velocity of the single dimer. This is the signature of the formation of large flattened swarms [28], with a representative structure shown in Fig. 6c. Note that the averages of $v_c/|v_s|$ and $\langle \mathbf{n}_i \cdot \mathbf{n}_j \rangle$ in Fig. 7 are underestimated, since they consider all clusters of a certain size, namely random clusters formed as a consequence of random collisions and aligned structures formed as consequence of the combination of phoretic and hydrodynamic interactions. We refer to this then as the *large swarms regime*. For the largest densities, the collisions between emergent fronts and their quick dissolution dominate the dynamics of the system. At the volume fraction $\phi =0.1$ in Fig. 6d, huge cluster sizes are reached, but the cluster velocities analyzed in Fig. 7 are very low and the orientational correlation is close to zero. This indicates that the clusters formed are dominated by just frequent encounters of dimers with different orientations, similar to the clusters formed with motility-induced separation [63, 65], which are here very unstable. This is a liquid-like state, and we refer to it then as the *dynamic jamming regime*.

**Fig. 8:**
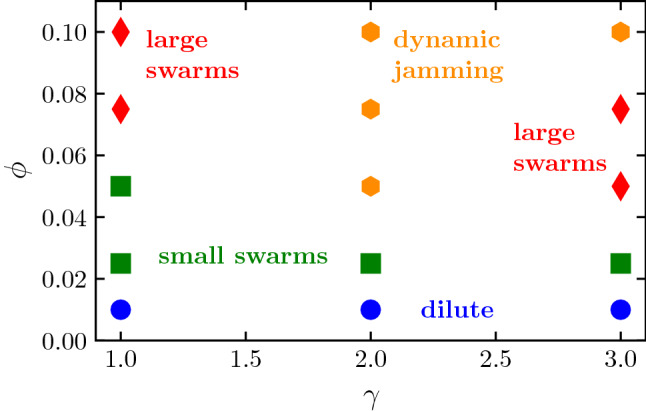
Dynamical state diagram of thermophobic dimers as a function of their density and bead aspect ratio $\gamma$. Symbols colors differentiate the dynamical states corresponding to the systems analyzed in Fig. 7

The asymmetric dimer with $\gamma =2$ shows very similar behavior to the one just described for $\gamma =3$. The flow fields of the two single asymmetric dimers are very similar, as can be seen in Figs. 1 and 2b, although the lobe leading to long-ranged lateral attraction is less prominent for $\gamma =2$, which weakens the hydrodynamic lateral attraction. Although the dynamic behavior is in principle similar, the stability of the clusters is reduced. This makes the jamming state start at lower densities, and the large swarming state disappears for the analyzed cases, as can be seen in Fig. 7. The symmetric dimer with $\gamma =1$ shows a different hydrodynamic flow field and behavior than the asymmetric dimers, see Figs. 1 and 2b, mainly long-ranged lateral hydrodynamic repulsion, but for a short-ranged attractive part, a different collective behavior could be expected. Ensembles of symmetric thermophobic dimers show a dilute phase at low volume fractions, and a small swarms state for slightly larger densities, same as for the asymmetric dimers. Interestingly, the small swarm state is present for densities clearly larger than for the asymmetric dimers, here up to $\phi =0.05$, with almost no difference in cluster maximum and average size, or the cluster velocity and orientation, besides the statistical noise of the measurements. For considerably larger densities, here up to $\phi =0.10$, the system presents all the features of the large swarm regime, with clusters of about 40 to 60 dimers with larger orientations and $v_c$ of about $25\%$ the velocity of the single dimer. Even larger volume fractions have not been tested, although it is to be expected that a dynamic jamming state will be observed. Since the long-ranged hydrodynamic interactions are repulsive in this case, the stability of the flattened structures can only stem from the short-ranged part of the hydrodynamic interactions, which are attractive for pairs of dimers whose phoretic beads surfaces are separated less than $s_\mathrm {p}$, as can be seen in Fig. 2b. A dynamical state diagram summarizing the results for the different symmetries and densities is shown in Fig. 8.

Important to note is that all simulation results here shown correspond to a fixed large value of the colloid surface temperature, and a particular choice of solvent–colloid potentials which determines the thermophoretic interaction strengths. This implies that, in contrast to other theoretical studies, the Péclet number is not the same for different structures, namely different values of $\gamma$. We remind here that in the case of phoresis, the Péclet number by itself is not enough to describe the system since it does not account for the intensity of the repulsion (attraction in the thermophilic case), which strongly influences the system behavior. Our approach (fixed potential and fixed temperature difference) corresponds to an experimental setup in which beads of two given materials are provided to build the dimers, such as gold and silica [6], and only one laser intensity is used. To vary the laser intensity would translate in different Péclet numbers, which would be again different for dimers with different $\gamma$ values. Decreasing the laser intensity decreases the temperature gradients, and this makes not only that the dimers velocity is smaller but simultaneously this also diminishes the inter-dimers phoretic repulsion, and the intensity of the hydrodynamic interactions, which are expected to remain qualitatively the same. The location of the different dynamical states might then be displaced, but will remain essentially the same as here described, just changing gradually to the equilibrium states for small enough values of the Péclet number. The approximation of disregarding shadowing effects here taken is not expected to be dramatic given the reasonably dilute and dynamic structures here discussed.

## Summary and conclusions

Thermophoretic dimeric active colloids are here investigated by means of hydrodynamic simulations. The aspect ratio of the radius of the two beads and the thermophilic or thermophobic character of the dimers modifies not only the single particle but also the collective behavior. The induced flow field around single dimers is pusher-type for symmetric thermophilic dimers, as well as for asymmetric thermophobic ones. Reversely, asymmetric thermophilic dimers and symmetric thermophobic dimer have a puller-type-induced flow field. The flow fields can be related to the standard pusher and puller flow fields, but are essentially different from those, especially in the short-ranged hydrodynamics. In the collective regime, thermophilic dimers show to nucleate in large ordered static clusters, with the hot bead pointing outward, *i.e.*, against the swimming direction. This is in contrast to many other non-phoretic swimmers which cluster just due to propulsion and steric interactions. The combination of phoretic attraction and hydrodynamic interactions stabilizes the clusters of thermophilic active dimers in all the investigated geometries. Clusters of asymmetric thermophilic dimers are more spherical than those of symmetric ones. Thermophobic swimmers in the collective regime are completely different; in this case, dimers propel with the hot bead at the back and do not form any stable compact structure. However, the combination of propulsion, phoretic repulsion, and hydrodynamic attraction results, in some cases, in the formation of short-lived aligned swarming structures. The case of self-thermophobic dimers constitutes also a very interesting example for a swimmer whose collective behavior is not just quantitatively influenced, but qualitatively determined by both the short- and the long-ranged hydrodynamic interactions. Further theoretical and experimental investigations will help to clarify the role of hydrodynamic interactions and to extend the conclusions of this study to not only to a larger parameter range but also to shapes with different properties, such as trimeric or multimeric active colloids. Possible applications of phoretic active colloids will definitely broaden with this knowledge, offering then promising perspectives, for example, in the design of bio-compatible micromotors.
